# Proteomic Profiling of Donkey Milk Exosomes Highlights Bioactive Proteins with Immune-Related Functions

**DOI:** 10.3390/ijms26072892

**Published:** 2025-03-22

**Authors:** Yihong Liu, Qingshan Ma, Muhammad Zahoor Khan, Menghan Wang, Fokun Xiang, Xinyue Zhang, Xiyan Kou, Shuhuan Li, Changfa Wang, Yan Li

**Affiliations:** College of Agriculture and Biology, Shandong Engineering Technology Research Center for Efficient Breeding and Ecological Feeding of Black Donkey, Liaocheng University, Liaocheng 252000, China; 15037232776@163.com (Y.L.); horsegreenhill@163.com (Q.M.); zahoorkhattak91@yahoo.com (M.Z.K.); 15895837828@163.com (M.W.); 2022404234@stu.lcu.edu.cn (F.X.); 2022404192@stu.lcu.edu.cn (X.Z.); kxy19990113@163.com (X.K.); 2024407099@stu.lcu.edu.cn (S.L.)

**Keywords:** donkey milk, differentially expressed exosomal proteins, proteomics, immune system

## Abstract

The growing recognition of the role of milk-derived exosomes in metabolic and immunological processes has brought attention to the potential utility of donkey milk. However, the efficacy and bioactive components of donkey milk are underexplored. This study aimed to elucidate the proteomic profiles of exosomes isolated from donkey colostrum and mature milk using advanced four-dimensional (4D) label-free quantitative proteomics. A comprehensive analysis identified and quantified a total of 2293 exosomal proteins from donkey milk, including 276 differentially expressed exosomal proteins (DEEPs). The results revealed marked proteomic differences between colostrum and mature milk exosomes, particularly in proteins associated with immune responses and metabolic pathways. Exosomal proteins derived from colostrum were found to be enriched in immune-modulatory factors and glycan-related pathways, which may contribute to the enhancement in neonatal immune system development. In contrast, exosomal proteins from mature milk were predominantly associated with metabolic processes and cellular senescence. Protein–protein interaction (PPI) analysis further suggested that specific exosomal proteins highly expressed in colostrum could serve as nutraceutical components with potential health benefits for humans. In conclusion, this study underscores the distinct proteomic features and potential physiological roles of exosomes from donkey colostrum versus mature milk.

## 1. Introduction

Historically, donkey milk has been valued not only as a nutritional resource but also as a therapeutic agent across diverse cultures. Its recent surge in popularity is driven by the increasing recognition of dietary therapy, alongside its rich nutritional profile and bioactive functional properties [1]. Recognized for its potential health-promoting properties, donkey milk has been associated with immune system enhancement and anti-inflammatory effects [2]. Recent scientific investigations have begun to explore its possible applications in the management of various health conditions, including allergies, dermatological disorders, and metabolic diseases, underscoring its relevance in contemporary nutritional science and medical research [3,4,5]. Despite its longstanding use and promising attributes, research into the specific functional components and mechanisms of action in donkey milk remains in its early stages.

Donkey milk is composed of various nutrients, including proteins, fatty acids, vitamins, and minerals, as well as bioactive compounds such as immunoglobulins and lysozyme [6]. Notably, recent research has identified the presence of extracellular vesicles (EVs), or vesicle-like nanoparticles (VLNs) in donkey milk, which are posited to play significant roles in promoting health [7]. These dietary EVs are membrane-bound nanostructures ranging in size from approximately 80 to 300 nm, capable of encapsulating a variety of biomolecules, including lipids, RNAs, and proteins [8,9]. These biomolecules are integral to various health-promoting functions, such as modulating immune responses and supporting gut health [8,10]. Among EVs, exosomes are a specific subtype, typically sized between 30 to 150 nm, known for their role in mediating intercellular communication [11]. Emerging data suggest that the composition and functional properties of milk-derived extracellular vesicles (MEVs) are influenced by lactation stage, with notable variations observed between colostrum and mature milk [12]. While prior proteomic analyses of donkey milk across different stages have been conducted [13], a comprehensive understanding of the protein profile and the functional significance of its exosomes remains a gap in the current knowledge. Due to their unique cargo and biological roles, exosomes in donkey milk are increasingly recognized as key mediators of its bioactive properties, thus warranting further in-depth proteomic investigation.

The aim of this study is to utilize 4D label-free quantitative proteomics to investigate the proteomic variations between exosomes isolated from donkey colostrum and mature milk. This advanced analytical approach is especially well suited for detecting subtle but important differences in protein composition and functionality at various stages of lactation. By characterizing these differences, this research seeks to establish a theoretical framework for understanding the physiological roles of donkey milk. Ultimately, the findings of this study are expected to contribute to an enhanced understanding of the bioactive properties of donkey milk, with potential implications for its application in nutrition and dairy science.

## 2. Results

### 2.1. Extraction and Characterization of Exosomes from Donkey Milk

**Figure 1:**
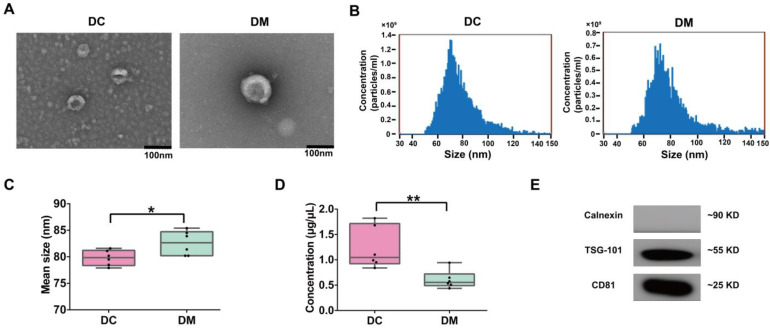
Characterization of DC and DM exosomes. (**A**) TEM observation of exosomes (scale bar = 100 nm). (**B**) Particle diameter distribution was analyzed by NanoFCM (representative data). (**C**) Box plots of mean size (diameter) of DC and DM exosomes. (**D**) Box plots of protein concentration in DC and DM exosomes. (**E**) WB analysis of exosomes markers CD81 and TSG101 and negative marker Calnexin. DC, donkey colostrum; DM, donkey mature milk. * *p* < 0.05, ** *p* < 0.01.

Exosomes were isolated from donkey colostrum and mature milk and subsequently characterized using TEM, NanoFCM, and WB (Figure 1). TEM analysis demonstrated that the exosomes exhibited a typical round or cup-shaped morphology, with an average diameter of approximately 80 nm (Figure 1A). The size distribution analysis revealed that exosomes from colostrum were significantly smaller (79.8 ± 0.59 nm) compared to those from mature milk (82.6 ± 0.93 nm, *p* < 0.05) (Figure 1B,C). Notably, the protein concentration of colostral exosomes was significantly higher than that of mature milk exosomes (Figure 1D). The analysis conducted through WB validated the existence of particular exosomal marker proteins, such as CD81 and TSG101, within exosomes found in both colostrum and mature milk. (Figure 1E and Figure S1).

### 2.2. Proteomic Profiling of Exosomal Proteins from Donkey Milk

**Figure 2:**
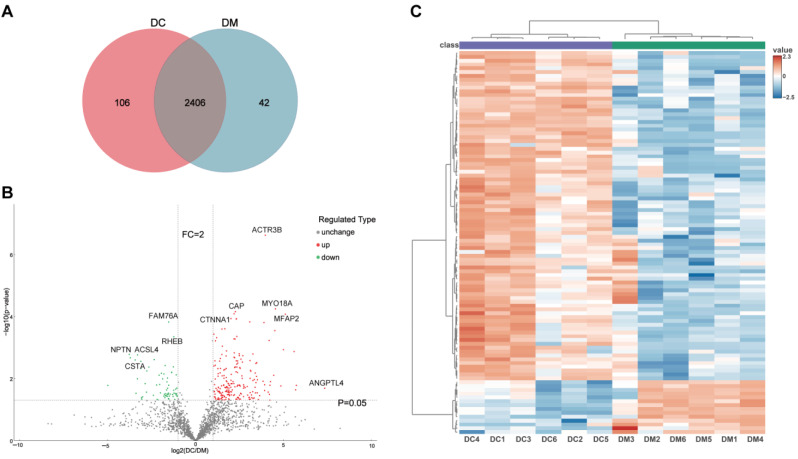
Differentially expressed exosomal proteins profile derived from DC and DM. (**A**) Venn diagram of the identified proteins in DC and DM exosomes. (**B**) Volcano plots showing the differentially expressed exosomal proteins from DC and DM. (**C**) Hierarchically clustered heatmaps of the differentially expressed exosomal proteins. DC, donkey colostrum; DM, donkey mature milk; FC, fold change.

To comprehensively investigate the proteomic differences between exosomes derived from donkey colostrum and mature milk, a four-dimensional (4D) label-free quantitative proteomics approach was utilized. The quality control results of the mass spectrometry analysis are presented in Figure S2. In total, 16,197 peptides were identified, corresponding to 14,966 unique peptides. These peptides were mapped to 2555 protein groups, of which 2293 contained quantifiable data (Figure S2A). Comparative analysis revealed that 2406 proteins (94.2%) were shared between both groups, whereas 106 proteins (4.1%) were uniquely detected in colostrum exosomes, and 42 proteins (1.6%) were specific to mature milk exosomes (Figure 2A). Among these, 276 DEEPs were identified by comparing the protein expression between colostrum exosomes and mature milk exosomes, with 211 proteins being up-regulated and 65 proteins down-regulated (Figure 2B and Table S1). Principal component analysis (PCA) and hierarchical clustering heatmaps illustrated distinct proteomic profiles of exosomes derived from colostrum versus mature milk, highlighting significant differences in protein expression patterns (Figure S2E and Figure 2C).

### 2.3. GO Annotation and KEGG Enrichment Analysis of DEEPs in Donkey Colostrum and Mature Milk

**Figure 3:**
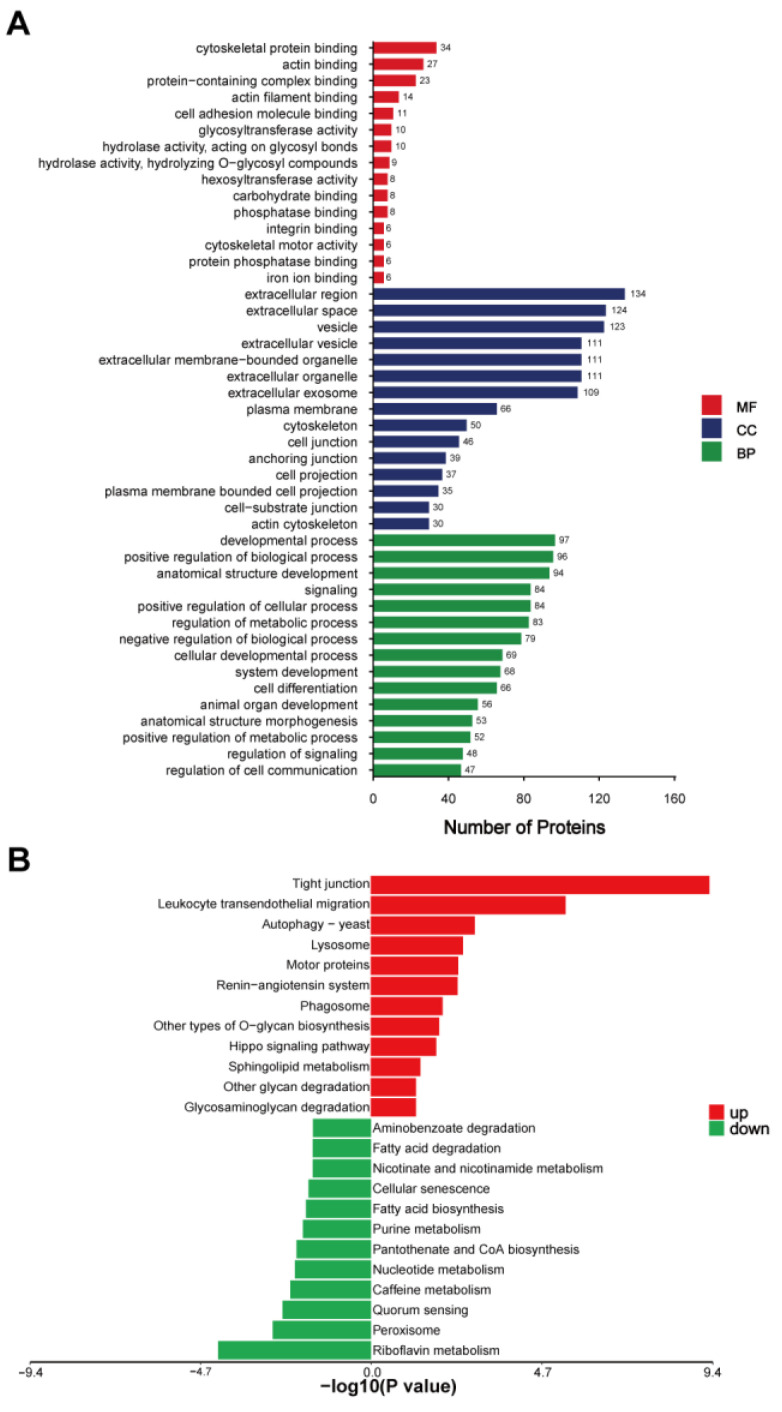
(**A**) GO annotation of the differentially expressed exosomal proteins. (**B**) KEGG pathway enrichment analysis of the differentially expressed exosomal proteins, DC/DM. DC, donkey colostrum; DM, donkey mature milk; MF, molecular functions; CC, cellular components; BP, biological processes.

To better understand the functional roles of the DEEPs identified between donkey colostrum and mature milk, we conducted comprehensive GO annotation and KEGG pathway enrichment analyses (Figure 3 and Table S1). The GO annotation categorized the 276 DEEPs into three primary groups: cellular components (CCs), biological processes (BPs), and molecular functions (MFs) (Figure 3A). In the cellular component category, the most enriched terms included the extracellular region, extracellular space, vesicle, extracellular organelle, extracellular membrane-bound organelle, extracellular vesicle, extracellular exosome, plasma membrane, cytoskeleton, and cell junction. For biological processes, the most significant annotations were related to the developmental process, positive regulation of biological processes, and anatomical structure development, suggesting that these proteins may play roles in cell growth and differentiation. In terms of molecular functions, the most prevalent terms were related to cytoskeletal protein binding, actin binding, protein-containing complex binding, actin filament binding, and cell adhesion molecule binding, highlighting the involvement of these proteins in cellular structure and adhesion.

The KEGG pathway enrichment analysis provided additional insights into the biological pathways influenced by the DEEPs, revealing distinct patterns between colostrum and mature milk exosomes. The DEEPs that were upregulated in colostrum were predominantly associated with pathways involved in cellular communication and immune responses. Notably, proteins enriched in colostrum exosomes were involved in tight junctions (e.g., cortactin, catenin beta 1, claudin-7, claudin-3, and vasodilator-stimulated phosphoprotein), leukocyte transendothelial migration (e.g., alpha-actinin-4, thy-1 membrane glycoprotein, afadin, and moesin), and the autophagy-yeast pathway (e.g., actin-related protein 3B and actin-related protein 2/3 complex subunit 5-like). Additionally, pathways related to lysosomal function (e.g., sialidase-1, tissue alpha-L-fucosidase, and procathepsin L), motor proteins (e.g., myosin-XVIIIa, myosin-14, and myosin regulatory light chain 12B), and the renin–angiotensin system (e.g., aminopeptidase N-like and thimet oligopeptidase) were also enriched, suggesting an enhanced involvement in vesicle transport, immune modulation, and protein degradation. Phagosome-related pathways, featuring proteins like thrombospondin-4, V-type proton ATPase subunit G 1, and procathepsin L, further underscore the active role of colostral exosomes in immune defense mechanisms. In contrast, DEEPs enriched in mature milk exosomes predominantly mapped to metabolism-related pathways, indicating a shift in functional focus as lactation progresses. Metabolic pathways such as riboflavin metabolism (e.g., ectonucleotide pyrophosphatase/phosphodiesterase family member 3-like and flavin reductase (NADPH)), peroxisome (e.g., mitochondrial fission 1 protein and xanthine dehydrogenase/oxidase), and quorum sensing (e.g., acyl-CoA synthetase long chain family member 1 and member 4) were significantly represented. Additionally, pathways related to caffeine metabolism, nucleotide metabolism, pantothenate and CoA biosynthesis, purine metabolism, and fatty acid biosynthesis were notably enriched in mature milk exosomes, involving key enzymes such as xanthine dehydrogenase/oxidase and acyl-CoA synthetase long-chain family members (Figure 3B and Table S1).

### 2.4. Protein–Protein Interaction Network Analysis of DEEPs in Donkey Colostrum and Mature Milk

**Figure 4:**
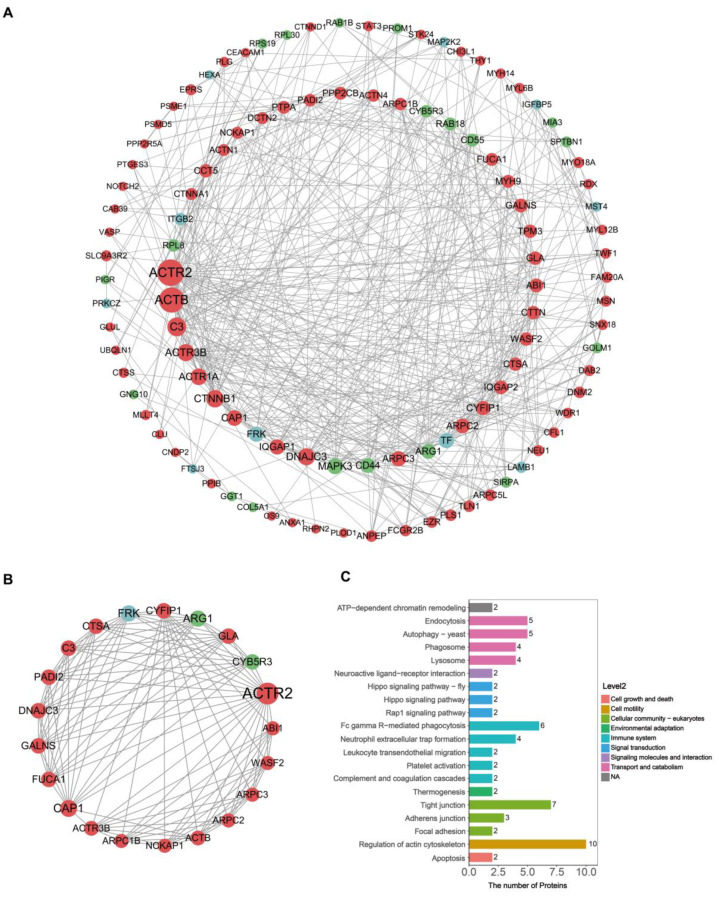
PPI network construction and cluster analysis. (**A**) PPI network for differentially expressed exosomal proteins. Proteins with a degree below 5 are hidden; node size represents the degree, with red denoting up-regulated proteins and green denoting down-regulated proteins. (**B**) The top 1 cluster (score = 11.5, nodes = 21, edges = 230). (**C**) KEGG pathway analysis of proteins in Cluster 1.

The DEEPs identified in donkey colostrum and mature milk were subjected to protein–protein interaction (PPI) network analysis using the STRING and Cytoscape software (version 3.9.1). The PPI network constructed consisted of 240 nodes and 1446 edges (Figure 4). The top 10 hub proteins in network in order were actin-related protein 2 (ACTR2), actin, cytoplasmic 1 (ACTB), complement C3 (C3), actin-related protein 3B (ACTR3B), alpha-centractin (ACTR1A), catenin beta-1 (CTNNB1), adenylyl cyclase-associated protein 1 (CAP1), ras GTPase-activating-like protein IQGAP1 (IQGAP1), dnaJ homolog subfamily C member 3 (DNAJC3), and mitogen-activated protein kinase 3 (MAPK3) (Figure 4A). Of these ten hub proteins, nine were found to be upregulated in colostral exosomes, while one was downregulated. Subsequent analysis focused on identifying key interconnected clusters within the PPI network. Cluster 1, which had the highest MCODE score (score > 10), was highlighted for further investigation (Figure 4B). This indicates that the proteins in Cluster 1 may play a crucial role in regulating the PPI network. Following this, we conducted a KEGG pathway analysis of the proteins within Cluster 1, identifying several proteins associated with the immune system, including ACTB, C3, ACTR2, ACTR3B, actin-related protein 2/3 complex subunit 1B (ARPC1B), actin-related protein 2/3 complex subunit 2 (ARPC2), ARPC3, and Wiskott–Aldrich syndrome protein family member 2 (WASF2) (Figure 4C and Table S2). Notably, these immune-associated proteins were upregulated in colostral exosomes, suggesting their potential importance as key regulators in the early establishment of the infant immune system.

## 3. Discussion

This study employed a proteomic-based approach to compare the proteomic profiles of exosomes from donkey colostrum and mature milk. Previous studies have characterized exosome proteomes from various milks, including camel [14], goat [15], bovine [16], and buffalo [17]. For instance, Joshi et al. (2024) identified 331 common exosomal proteins across three buffalo milk samples [17]. In this study, 2406 proteins (94.2%) were consistently identified in both donkey colostrum and mature milk exosomes. Significant differences in exosome proteomes have been noted across lactation stages and species [18]. Our results revealed proteomic differences between colostrum and mature milk exosomes, with 211 proteins upregulated and 65 downregulated in colostrum. Specifically, immune-related proteins such as complement C1q tumor necrosis factor-related protein 3 (C1QTNF3; FC > 50), sialidase-1 (NEU1; FC > 19), and glutathione peroxidase 3 (GPx3; FC > 15) were upregulated in colostrum. These proteins have been consistently associated with critical biological function processes. For instance, C1QTNF3 regulates inflammatory pathways [19], NEU1 modulates cellular signaling and shows therapeutic potential for cancer and immune disorders [20], and GPx3 is known for its antioxidant and anti-inflammatory properties [21]. Notably, we found angiopoietin-related protein 4 (ANGPTL4) to be significantly increased in colostrum, exhibiting a remarkable 163-fold increase compared to mature milk. ANGPTL4 is recognized for its regulatory roles in lipid metabolism and glucose metabolism [22], angiogenesis [23], and inflammation [24]. Interestingly, the presence of ANGPTL4 at such elevated levels in milk has not been extensively reported in the literature, especially in the context of colostrum. This novel observation highlights the specialized role of colostrum in supporting neonatal health. Further investigations are warranted to elucidate the potential contributions of ANGPTL4 to metabolic regulation and immune adaptation in neonates, potentially informing strategies for functional nutrition and neonatal care.

The KEGG pathway analysis of DEEPs revealed that proteins upregulated in donkey colostrum are primarily enriched in pathways related to autophagy, phagosome, and lysosome activity. This suggests a pivotal role for these pathways in providing early immune protection and facilitating cellular clearance in newborns. These findings align with previous research indicating that autophagosomes can merge with phagosomes to enhance the degradation of apoptotic cells, thereby accelerating immune defense mechanisms [25]. The enhanced activity of these pathways in colostrum may offer newborns enhanced immune protection during the critical early stages of life, contrasting with mature milk, which prioritizes long-term nutritional support rather than acute immune defense. Previous research, particularly in bovine milk, has shown that exosomes are typically degraded by lysosomes, which may limit their functional biological activity [26]. However, our data suggest that in colostrum, there is an increased involvement of autophagy and phagosome–lysosome fusion processes. This enhanced activity likely reflects the unique bioactivity of colostrum in promoting cellular recycling and immune modulation, underscoring its specialized role beyond mere nutrition in neonatal health. These insights expand our understanding of colostrum’s contributions to early immune regulation, highlighting its critical function in neonatal development. Additionally, our analysis identified significant enrichment of pathways related to O-glycan biosynthesis, glycan degradation, and glycosaminoglycan degradation in colostrum. These glycan-associated pathways play essential roles in immune modulation, pathogen recognition, and the development of gut microbiota, functions that have been underreported in previous milk proteomics studies. O-glycan biosynthesis is crucial for the production of glycoproteins, including immune molecules like IgA, which protect the mucosal surfaces of neonates from pathogens [27]. Moreover, the glycan degradation pathways in colostrum provide substrates for beneficial gut bacteria, supporting the establishment of a healthy intestinal microbiome in newborns [28]. These pathways likely contribute to early immune defense, extracellular matrix remodeling, and tissue repair, which are essential during the rapid growth phase of neonates. In contrast, the proteomic profile of mature milk exosomes showed significant enrichment in metabolism-related pathways, highlighting their crucial role in supporting energy metabolism and infant development. Notably, several key proteins involved in lipid metabolism were highly expressed in exosomes from mature milk, including Acyl-CoA synthetase long-chain family member 4 (ACSL4; FC > 9), Acyl-CoA synthetase long-chain family member 1 (ACSL1; FC > 8), and perilipin 2 (PLIN2; FC > 2), which is line with findings previously reported [13]. The critical role of ACSL4 and ACSL1 in the regulation of fatty acid metabolism has been well documented [29,30]. In addition, perilipin 2 has been found to be involved in the formation and storage of lipid droplets [31,32]. Collectively, these proteins contribute significantly to the synthesis and storage of milk lipids, which are essential for the growth and development of infants. Moreover, a notable enrichment of markers associated with cellular senescence was observed in exosomes from mature milk. This suggests that mature milk exosomes might play a role in immune modulation and tissue homeostasis for both mothers and infants. The presence of senescence-associated secretory phenotype (SASP) factors in these exosomes could influence developmental processes [33]. These findings open new avenues for understanding the functional differences in exosomal content across lactation stages and warrant further investigation to elucidate their implications for neonatal health and development.

PPI network analysis identified the top 10 nodal proteins, which include ACTR2, ACTB, C3, ACTR3B, ACTR1A, CTNNB1, CAP1, IQGAP1, DNAJC3, and MAPK3. With the exception of MAPK3, the other nine nodal proteins are upregulated in donkey colostrum exosomes. Through the KEGG enrichment analysis of Cluster 1, we found that the proteins associated with the immune system include ACTB, C3, ACTR2, ACTR3B, ARPC1B, ARPC2, ARPC3, and WASF2. ACTB is essential for cell motility and morphology, significantly influencing the migration of leukocytes to infection sites [34]. C3 facilitates opsonization, thereby enhancing the phagocytosis of pathogens and triggering inflammation [35]. The ACTR2 and ACTR3B proteins are integral components of the Arp2/3 complex, promoting actin polymerization, a process vital for dendritic cell function and T cell activation [36]. Additionally, ARPC1B, ARPC2, and ARPC3 are also a part of the Arp2/3 complex and play a crucial role in coordinating the actin cytoskeleton during immune responses, thereby enhancing cell–cell interactions and antigen presentation [37,38]. Furthermore, WASF2 is involved in actin polymerization, promotes T cell receptor signaling, and enhances immune responses [39]. Through the interaction network analysis of these DEEPs, we speculate that these proteins play a significant role in the immune response. Certain exosomal proteins, particularly those derived from donkey colostrum, may serve as nutraceuticals or dietary supplements in human nutrition, potentially enhancing health and resistance. Notable examples of these proteins include ACTB, C3, ACTR2, ACTR3B, ANGPL4, and other relevant components. Although the precise functions of individual proteins and the significance of specific pathways require further elucidation, these findings offer valuable insights into the functional properties of donkey milk exosomes. Future research should not only validate protein efficacy through in vivo and in vitro studies but also address the limitations associated with pooled samples by analyzing individual samples to more accurately capture biological variability. A key factor influencing the bioactivity of exosomal proteins is their stability during gastrointestinal digestion, as their functional significance depends on their ability to withstand enzymatic degradation and reach target sites intact. Studies have demonstrated that milk-derived exosomes exhibit notable resistance to gastric digestion due to their protective lipid bilayer [40,41], which shields their protein and RNA cargo. Moreover, exosome-encapsulated proteins show greater stability and bioavailability compared to free counterparts [42], potentially enhancing their functional efficacy. Given these properties, future studies should further investigate the digestive stability and absorption mechanisms of donkey milk exosomes to elucidate their comprehensive nutritional and therapeutic potential. Nevertheless, this study also contributes to a deeper understanding of the biological functions of donkey milk, potentially increasing its recognition and appeal as a health-promoting dietary component.

## 4. Materials and Methods

### 4.1. Animals and Sample Collection

Thirty female Dezhou donkeys (in their second parity), aged 4 to 5 years, were selected for colostrum (1–3 days postpartum) and mature milk (30 days postpartum) sample collection at the National Black Donkey Breeding Center in Liaocheng City, China. Prior to sampling, the foals were separated from their mothers for 3 h but were kept in an adjacent stall. Milk samples were collected using a standard hand-milking technique by the same farmer and immediately transported to the laboratory without delay using dry ice, where they were stored at −80 °C. For each lactation stage, five individual milk samples from both colostrum and mature milk were pooled into a single composite sample, resulting in six biological replicates per stage. All animals were raised under uniform conditions and provided with the same feed. The animal care procedures in this study adhered to standard commercial management practices and were approved by the Animal Welfare Committee of Liaocheng University (Permit No. AP2024061227).

### 4.2. Isolation and Characterization of Exosomes from Donkey Milk

The extraction of exosomes from donkey milk was performed following the protocol of Gupta et al. (2018), with appropriate modifications [43]. Ultracentrifugation on a sucrose cushion was utilized for the purpose of isolating and purifying exosomes. Initially, milk samples underwent two sequential centrifugation steps (2000× *g* for 30 min at 4 °C and 10,000× *g* for 45 min at 4 °C). The supernatant obtained was filtered through a 0.45 μm membrane filter, and the resulting filtrate was subsequently placed into a fresh tube for ultracentrifugation (100,000× *g* for 70 min at 4 °C). Following the removal of the supernatant, the sediment was resuspended in 1 mL of pre-cooled 1×PBS. The resuspended exosomes (1 mL) were added to a 30% sucrose cushion (250 μL) and subjected to ultracentrifugation once more at 100,000× *g* for 70 min. After centrifugation, 250 μL of the resulting solution was gathered, diluted with PBS to reach a total volume of 3 mL, and subjected to a final ultracentrifugation step (100,000× *g* for 70 min). The supernatant was removed, and the exosome pellet was carefully resuspended in 500 μL of phosphate buffer before being stored at −80 °C. The characterization of exosomal protein concentration, morphology, and particle size was performed through the use of a BCA protein assay (Bio-Rad, Hercules, CA, USA), transmission electron microscopy (TEM; HT-7700, Hitachi, Tokyo, Japan), and a nanoflow cytometer (NanoFCM, N30E, Xiamen, China). Additionally, exosome marker proteins were analyzed by Western blot (WB), targeting Calnexin (Signalway Antibody, College Park, MD, USA), CD81 (Santa Cruz Biotechnology, Santa Cruz, Dallas, TX, USA) and TSG101 (Novus Biologicals, Littleton, CO, USA).

### 4.3. Protein Extraction and Digestion

Exosomal proteins were extracted and subjected to tryptic digestion utilizing a modified filter-aided sample preparation (FASP) technique, as outlined by Wiśniewski (2019) [44]. Initially, 50 μL of the sample was mixed with 20 μL of SDT buffer (4% SDS, 100 mM Tris-HCl, pH 7.6) and heated at 95 °C for 5 min. Once the mixture reached room temperature, 200 μL of 8M urea (UA) buffer was incorporated. Subsequently, it was transferred into a 10 kDa ultrafiltration centrifuge tube and subjected to centrifugation at 14,000× *g* for 30 min. The filtrate was discarded, and the washing process was repeated by adding another 200 μL of UA buffer before centrifugation (14,000× *g*, 30 min). Next, 100 μL of a 100 mM iodoacetamide solution was introduced into the ultrafiltration tube and incubated in the dark at room temperature for 30–60 min. The sample was subsequently rinsed two times using 100 μL of 8 M UA solution and then centrifuged (14,000× *g*, 25 °C, 30 min). This was followed by two more rinses with 100 μL of 25 mM ammonium bicarbonate (ABC) solution, also centrifuged under the same conditions. For protein digestion, 46 μL of trypsin buffer (which contains 1 μg of trypsin in 40 μL of 25 mM ABC solution) was added, and the mixture was allowed to incubate overnight at 37 °C. On the following day, the sample underwent centrifugation (14,000× *g*, 25 °C, 30 min) and was collected. An additional 40 μL of 25 mM ABC solution was added; the final sample was obtained through centrifugation (14,000× *g*, 25 °C, 30 min). Peptide desalting was carried out utilizing C18 cartridges (Empore SPE Cartridges C18, standard density, bed I.D. 7 mm, volume 3 mL, Sigma, Burlington, MA, USA). The desalted peptides were freeze-dried and re-dissolved in 40 μL of 0.1% formic acid for the following LC-MS/MS analysis.

### 4.4. LC-MS/MS Analysis

The analysis using LC-MS/MS was carried out utilizing a timsTOF Pro mass spectrometer, integrated with a Nano Elute system (Bruker, Bremen, Germany). Buffer A consisted of a 0.1% formic acid aqueous solution, while Buffer B was prepared as a 0.1% formic acid solution in acetonitrile, with acetonitrile with a purity of 99.9%. Prior to sample injection, the chromatographic column was equilibrated with 95% of Buffer A. For separation, a C18 reverse-phase column (Thermo Scientific EASY column, 25 cm length, 75 μm inner diameter, 1.9 μm particle size) was employed, operating at a flow rate of 300 nL/min. The elution process involved a programmed gradient of Buffer B. Initially, the concentration of Buffer B rose steadily from 0% to 40% over 48 min. Subsequently, there was a swift linear ascent from 40% to 90% in just 2 min. Finally, Buffer B was maintained at 90% for a duration of 10 min, allowing for thorough elution of the retained compounds. The analysis of precursor ions and their fragments was conducted using the time-of-flight (TOF) detector, with the MS/MS scan range spanning from 100 to 1700 *m*/*z*. Data acquisition utilized the parallel accumulation–serial fragmentation (PASEF) mode, optimizing sensitivity and speed.

### 4.5. Protein Identification and Quantification

The acquired MS/MS data from each run were analyzed using MaxQuant software (version 1.6.14) for comprehensive database searching and quantitative analysis [45]. Cysteine residues were fixed with carbamidomethylation (C), while methionine oxidation (M) was treated as a variable modification. The mass tolerances for the initial search and the main search were set at 20 ppm and 6 ppm, respectively, with an MS/MS mass tolerance of 20 ppm. The false discovery rate (FDR) was controlled using a target-decoy approach based on the Reverseprotein.fasta database, maintaining a threshold FDR of ≤0.01 for both peptide and protein identification. For quantification, relative intensity-based label-free quantification (LFQ) was utilized, employing a minimum ratio count of one to ensure reliable protein quantification.

### 4.6. Bioinformatic and Statistical Analysis

The R package MSstats was employed to obtain normalized peak areas for proteins. To visualize differentially expressed exosomal proteins (DEEPs), a *t*-test was conducted using R (version 3.4), which facilitated the generation of volcano plots and hierarchical clustering representations. The threshold for screening the DEEPs was set to *p* < 0.05 with a fold change of either >2.00 or <0.50. Hierarchical clustering heatmaps were created using the ComplexHeatmap R package (version 3.4). Gene Ontology (GO) annotations for the target proteins were performed using Blast2GO (version 2.8.0+) (https://geneontology.org/, accessed on 10 September 2024), while Kyoto Encyclopedia of Genes and Genomes’ (KEGG) pathway annotations were conducted using KEGG Orthology-Based Annotation System (KOBAS) (version 3.0) software (https://www.genome.jp/kegg/, accessed on 12 September 2024). Fisher’s exact test was utilized to assess the significance of GO and KEGG enrichment in DEEPs, applying a *p*-value threshold of <0.05, which is regarded as indicative of significant enrichment. The STRING database (http://stringdb.org/, accessed on 20 September 2024) was used to investigate both direct and indirect interactions among the target proteins, resulting in the generation of a protein–protein interaction (PPI) network map, which was visualized using Cytoscape (version 3.9.1). Following the visualization of the network with Cytoscape, densely connected protein modules within the PPI network were identified using the Molecular Complex Detection (MCODE) application with default parameter settings [46]. Moreover, KEGG enrichment pathway analysis for the top cluster was performed in accordance with the methods previously mentioned. Additionally, the donkey milk exosomes proteomic and MS raw data are publicly available from the iProX database (http://www.iprox.org, accessed on 17 March 2025, accession number: IPX0011399000).

## 5. Conclusions

This study provides a comprehensive analysis of the proteomic differences between exosomes derived from donkey colostrum and mature milk using 4D label-free quantitative proteomics. The findings highlight distinct proteomic profiles across lactation stages, indicating that colostral exosomes are enriched with immune-related proteins, particularly those involved in autophagy, phagosome formation, and lysosome pathways. In contrast, the exosomal proteins derived from mature milk are enriched in pathways related to nutritional metabolism. Additionally, certain exosomal proteins including ACTB, C3, ACTR2, ACTR3B, ANGPL4, and other relevant components, may significantly contribute to enhancing host health and immune resistance. Although we have not yet verified the precise functions of specific proteins and the critical roles of certain pathways, further investigation is essential for a more comprehensive understanding. These insights enhance our deep understanding of the biological functions of donkey milk and may increase public interest in its benefits.

## Data Availability

Data will be made available on request.

## Supplementary Materials

The following supporting information can be downloaded at: https://www.mdpi.com/article/10.3390/ijms26072892/s1.
