# Supplementary figures and images for: Proteomic Profiling of Donkey Milk Exosomes Highlights Bioactive Proteins with Immune-Related Functions
Source: Int J Mol Sci. 2025 Mar 22;26(7):2892. doi: 10.3390/ijms26072892 (PMC11988413; doi:10.3390/ijms26072892)

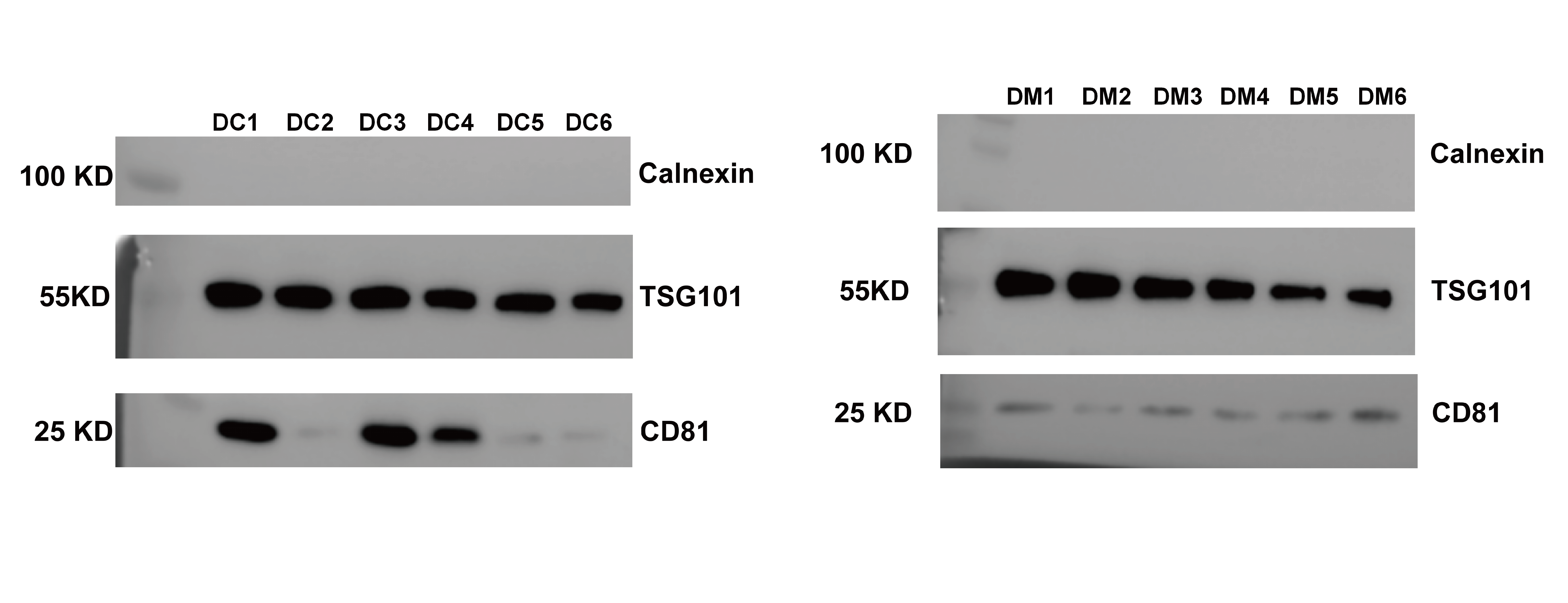

Supplement: Supplementary file 1 [file ijms-26-02892-s001.zip › Figure S1.tif]

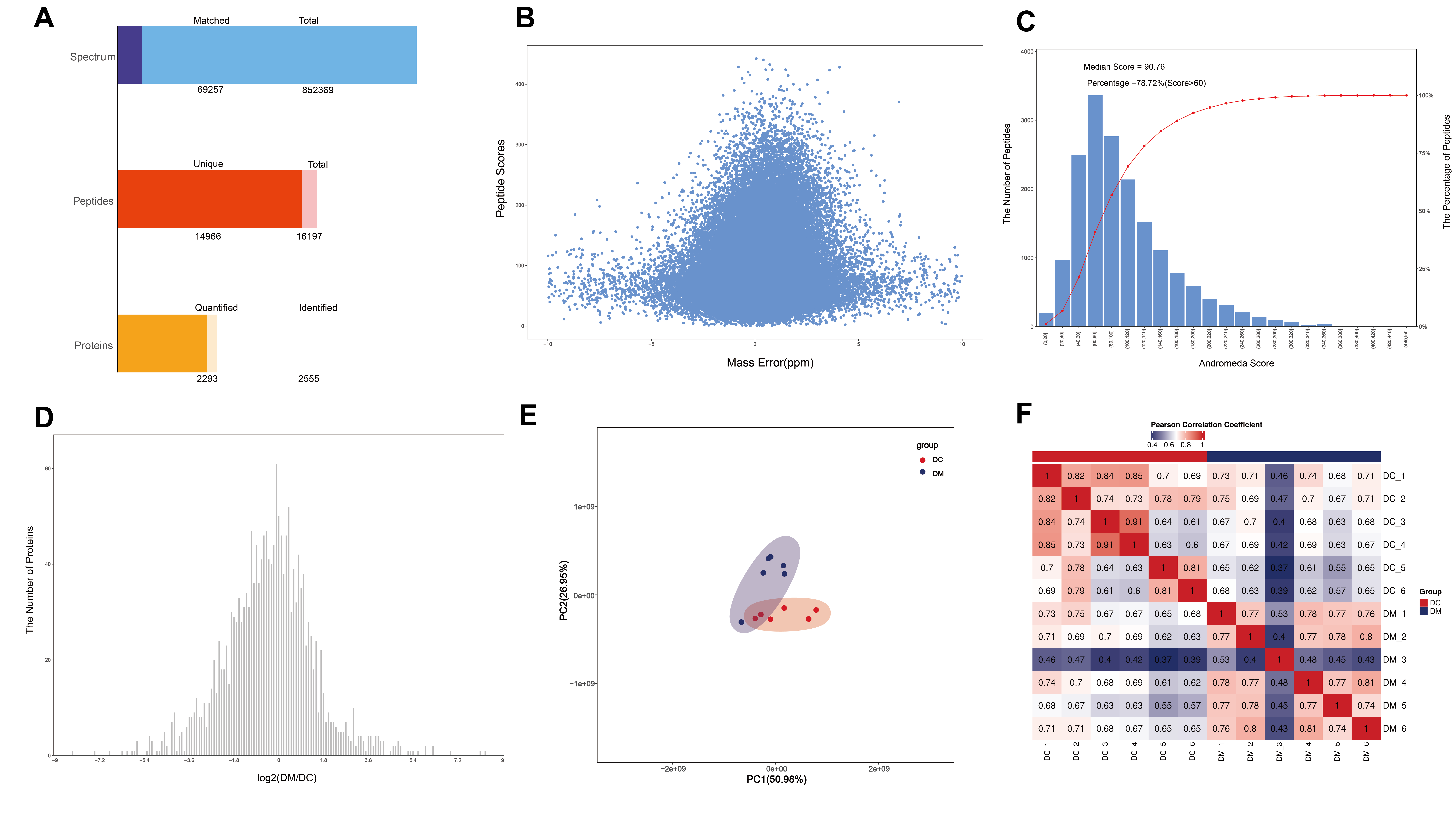

Supplement: Supplementary file 1 [file ijms-26-02892-s001.zip › figure S2.tif]
